# Early Prediction of Planning Adaptation Requirement Indication Due to Volumetric Alterations in Head and Neck Cancer Radiotherapy: A Machine Learning Approach

**DOI:** 10.3390/cancers14153573

**Published:** 2022-07-22

**Authors:** Vasiliki Iliadou, Ioannis Kakkos, Pantelis Karaiskos, Vassilis Kouloulias, Kalliopi Platoni, Anna Zygogianni, George K. Matsopoulos

**Affiliations:** 1 School of Electrical and Computer Engineering, National Technical University of Athens, 157 73 Athens, Greece; ioakakkos@gmail.com (I.K.); gmatso@esd.ece.ntua.gr (G.K.M.); 2 Department of Biomedical Engineering, University of West Attica, 122 43 Athens, Greece; 3 Medical Physics Laboratory, Medical School, National and Kapodistrian University of Athens, 115 27 Athens, Greece; pkaraisk@med.uoa.gr; 4 2nd Department of Radiology, Radiotherapy Unit, ATTIKON University Hospital, 124 62 Athens, Greece; vkouloul@ece.ntua.gr (V.K.); polaplatoni@gmail.com (K.P.); 5 1st Department of Radiology, Radiotherapy Unit, ARETAIEION University Hospital, 115 28 Athens, Greece; annazygo1@yahoo.gr

**Keywords:** head and neck cancer, radiation therapy, machine learning, CBCT, early prediction

## Abstract

**Simple Summary:**

Early prediction of significant alterations of head and neck (HN) cancer volume due to radiation therapy (RT) could provide an indication for necessary planning adaptations of the RT dose, tumor and organs at risk anatomy. However, the irregularities of the underlying cancer tissue and the patient-specific responses to RT render the prognostics for the tumor’s behavior exceedingly complex. In this study, a data-driven machine learning approach is proposed that incorporates the radiomic features of the low-dosage cone beam CT (CBCT) images clinically used in image-guided radiotherapy treatments in a feature selection and classification framework. The proposed model achieved high prediction performance, while able to identify indicative image characteristics for early prediction, further investigated in terms of their implications in HN cancer treated with RT.

**Abstract:**

Background: During RT cycles, the tumor response pattern could affect tumor coverage and may lead to organs at risk of overdose. As such, early prediction of significant volumetric changes could therefore reduce potential radiation-related adverse effects. Nevertheless, effective machine learning approaches based on the radiomic features of the clinically used CBCT images to determine the tumor volume variations due to RT not having been implemented so far. Methods: CBCT images from 40 HN cancer patients were collected weekly during RT treatment. From the obtained images, the Clinical Target Volume (CTV) and Parotid Glands (PG) regions of interest were utilized to calculate 104 delta-radiomics features. These features were fed on a feature selection and classification procedure for the early prediction of significant volumetric alterations. Results: The proposed framework was able to achieve 0.90 classification performance accuracy while detecting a small subset of discriminative characteristics from the 1st week of RT. The selected features were further analyzed regarding their effects on temporal changes in anatomy and tumor response modeling. Conclusion: The use of machine learning algorithms offers promising perspectives for fast and reliable early prediction of large volumetric deviations as a result of RT treatment, exploiting hidden patterns in the overall anatomical characteristics.

## 1. Introduction

Radiation Therapy (RT) is the primary treatment for Head and Neck (HN) cancer, deployed either on its own or as adjuvant treatment together with surgery and/or chemotherapy, allowing for high tumor control and cure rates based on a planning process that regulates the external radiation beams [1]. Although the treatment planning and dose accumulation calculations predominantly utilize planning Computed Tomography (pCT) images, additional information could be provided by images from other modalities, such as low-dosage Cone Beam CT (CBCT), therefore enhancing clinicians for treatment schemes decisions [2]. Such information can not only address spatial inaccuracies between the initial and the repetitive target positioning during treatment sessions but also highlight anatomical variations (such as weight loss and changes in tumor and organs at risk (OARs) volume, position and shape) that affect tumor coverage and OARs overdose [3,4]. On this premise, deviations between planned and delivered radiation dosage are reported to cause various adverse effects ranging from xerostomia to Parotid Glands (PGs) dysfunction, consequently affecting the patients’ wellbeing and thus underlying the pressing need for planning adaptations [5,6]. In fact, recent evidence suggests that planning adaptations are required during RT or between RT sessions, especially for patients who present more than 20–30% volumetric changes in parotid glands or Clinical Target Volume (CTV) [7,8]. To alleviate such anatomical differences, offline or online Adaptive Radiotherapy Treatment (ART) has been implemented, adapting the patient’s initial volumes and planning to the current anatomy and position [9,10]. However, ART’s complicated computations (where clinicians have to constantly analyze new scans and create new plans), significantly increase the overall workload and resource utilization burden, underlying the need for an efficient and cost-effective approach for the identification of the patients that will benefit from ART [11]. To that end, a large number of studies have investigated the statistical relationship between the need for ART and image-based characteristics [12,13]. Nevertheless, the development of early prediction multivariate models for ART application presents several challenges as a consequence of volume-related discrepancies, resulting from anatomical alterations in cancer patients treated with RT [14,15].

Since treatment planning adaptation requires non-invasive approaches to account for objective decision-making, image-based characteristics could provide robust measurements for anatomical changes. Changes in radiomic features during RT can be used as predictive factors, reflecting the tumor’s response to treatment and therefore offer effective predictive representation [16]. From this perspective, machine learning algorithms have proven to be valuable tools offering unique advantages in large multivariate data processing, while allowing robust modeling of HN cancer progression and RT treatment course outcomes [17,18]. More importantly, they can elucidate the complex relations between the multiple image-related features, providing an insight into the effects of RT, thus alerting clinicians to necessary treatment planning adaptations [19,20]. In this regard, they have been implemented in numerous radiation oncology applications, automatizing clinical procedures and improving auto-contouring efficiency, treatment planning, quality assurance, motion management and outcome predictions [21]. Furthermore, machine learning algorithms have demonstrated accurate prediction of tumor response to radiotherapy, prediction of radiation-induced toxicities and other side effects [22]. For instance, Zhang et al. [23] used diffusion kurtosis calculated from MRI images to predict radiotherapy effects for esophageal carcinoma, while Liu et al. [24] employed delta-radiomics on CT images for acute xerostomia early detection during RT for nasopharyngeal cancer achieving over 0.92 precision. To achieve that, patients were separated into classes based on the evaluation for the amount of acute xerostomia using the RTOG acute toxicity scoring and the salivary amount per case. Other research works have investigated significant prognostic features based on a radiomic analysis of 440 features for lung and HN cancer, revealing that tumor heterogeneity is correlated with poorer prognosis and that a more heterogeneous salivary gland texture could be associated with adverse treatment outcomes [25]. More recently, a study examined the characteristics extracted from contoured Regions of Interest (ROIs) and on Dose-Volume Histogram (DVH) information, suggesting that salivary glands radiomic features have the potential to predict late post-RT xerostomia beyond delivered radiation dose [26]. Taking into consideration all the above, it can be inferred that machine learning methods can be employed for the development of clinical support systems in order to provide early warnings of significant anatomical changes due to RT, assisting patients’ re-planning early in the course of radiation intervals.

However, limited research so far has investigated the feasibility of machine learning applications based on radiomic-derived features for the early prediction of significant tumor volume alterations necessitating adaptive radiotherapy. On that premise, Tanaka et al., 2022 [27] employed machine learning on HN cancer patients to early indicate ART. By incorporating radiomics features derived from CT images (one before the start of radiotherapy and one during radiotherapy for boost planning) in a deep learning design, they were able to obtain efficient predictive performance (Area Under the Curve = 0.73 to 0.75). In a similar fashion, Alves et al., 2021 [28] utilized sematic features, radiomic features (extracted from contrast-enhanced CT) and a combination of the two, to discriminate between replan (ART) and control (no-ART) groups by applying an SVM classifier. Their proposed design reported a radiomic-based mean accuracy of 0.78, with the feature combination further increasing classification performance (mean accuracy = 0.82). Following on from this, the scarcity of relevant research could be related to the fact that simple image features present limited discrimination ability for the successful assessment of the RT effects, probably due to the similarity of the soft tissues (tumor) and the normal (healthy) tissues surrounding the tumor limits. In fact, early RT complications (as soon as the 1st week of treatment) due to radiation-induced glandular hyperemia have been reported to affect gray values and, as a consequence, the calculated feature variables [29]. To that end, sonographic findings along with ultrasound clinical observations have reported disparities between pre- and post-radiotherapy PG image textures, likely as a result of fibrotic scarring of RT treatment [30]. The hypoxic microenvironment in HN squamous cell carcinoma areas has also demonstrated dynamic variations in CT texture characteristics, such as entropy, since it contains subpopulations of tumor cells exposed to the changing gradients of oxygen [31].

In this paper, we propose a CBCT radiomics-features machine-learning framework for the early prediction of significant anatomical alterations attributable to RT in HN cancer patients, indicating treatment planning adaptation Moreover, we focus on delineating the significant radiomics characteristics in terms of classification discriminative ability and their implications in the volumetric alterations early prediction. To the best of our knowledge, this is the first study to apply radiomics features in clinically used CBCT images for the early detection of tumor-volume significant deviations.

As such, we employ a Recursive Feature Elimination with Correlation Bias (RFE-CBR) feature selection procedure paired with Support Vector Machine (SVM) classifiers to predict anatomical alterations (significant anatomical changes are expected after the 3rd week of RT as indicated by our previous work [8]), utilizing characteristics derived by Clinical Target Volume (CTV) and Parotid Glands (PG) ROIs on CBCT images. The proposed multi-variable framework resulted in high predictive power (0.90 accuracy) from the 1st week of RT, while being able to identify a small set of image-based measures that can be indicative of temporal changes in anatomy providing a better overview for the application of ART.

## 2. Materials and Methods

### 2.1. Patients

The data were acquired from 40 HN cancer patients (25 male) with a mean age of 56.6 ± 11.2 years. All patients had the same histology, by means of squamous cell carcinoma of the HN, and were treated with volumetric modulated arc therapy (VMAT) with a prescription dose of 66 Gy in 30 fractions, over 6 weeks (5 sessions per week). For each patient, a standard clinical protocol for radiation treatment planning was applied, while the planning CT (pCT) images, the Dicom-RT structure file, and the CBCT scans of the first session per week (6 CBCT data sets) that were used for Image-Guided Radiation Therapy (IGRT) were recorded per patient. All data used were anonymized and exported in Digital Imaging and Communications in Medicine (DICOM) file format. Moreover, the contoured structures created for RT treatment planning were also anonymized and exported in DICOM-RT file format. The study was approved by the ATTIKON University Hospital, Athens, Greece relevant ethics committee (Protocol ΕΒΔ304/11-05-2022, 7 June 2022) in accordance with the Declaration of Helsinki. All data used were anonymized and exported in Digital Imaging and Communications in Medicine (DICOM) file format. Moreover, the contoured structures created for RT treatment planning were also anonymized and exported in DICOM-RT file format.

The study was conducted in accordance with the Declaration of Helsinki and approved by the Institutional Review Board. Patient consent was waived as all patient data were analyzed retrospectively after being anonymized. No additional images were acquired for the purposes of this study.

### 2.2. Data Pre-Processing and Class Designation

**Figure 1:**
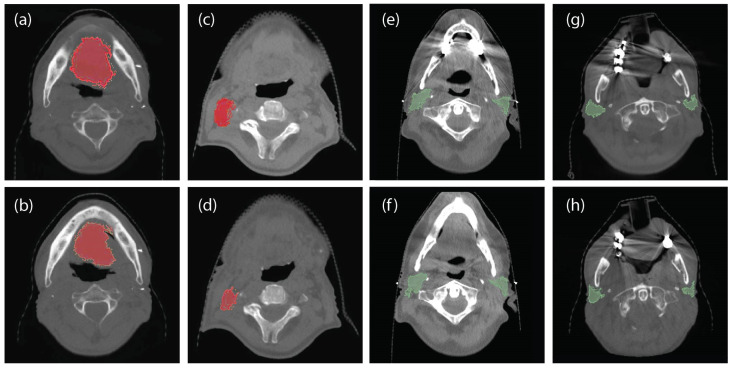
CBCT scans for patients at baseline (week 0) and at the end of the RT treatment sessions with significant and non-significant changes for the CTV tumor region (red mask) and the PG tumor region (green mask). Specifically: (**a**) CTV ROI of patient #3 at baseline; (**b**) CTV ROI of patient #3 at the end of the RT treatment sessions (non-significant changes); (**c**) CTV ROI of patient #14 at baseline; (**d**) CTV ROI of patient #14 at the end of the RT treatment sessions (significant changes); (**e**) PG ROI of patient #3 at baseline; (**f**) PG ROI of patient #3 at the end of the RT treatment sessions (non-significant changes); (**g**) PG ROI of patient #18 at baseline; (**h**) PG ROI of patient #18 at the end of the RT treatment sessions (significant changes).

As described above, each patient dataset incorporated a Dicom-RT structure file, a pCT image, and six (6) CBCT scans for IGRT obtained at the start of each week of treatment. After imaging data collection, a series of previously validated preprocessing steps took place to correct for position and shape irregularities [8]. Specifically, Rigid and Deformable image registration was used in order to align the initial planning-CT to the weekly CBCT images. Furthermore, each patient’s anatomical structure, designed in the initial planning-CT image, (i.e., the Clinical Target Volume (CTV) and Parotid Glands (PG)), were deformed based on the corresponding CBCT scans. Deformed Regions of Interest (ROIs) (CTV and PG volumes) were evaluated both visually and with the Mean Distance to Agreement metric as the level of conformity between the deformed and the assessed-by-an-expert ROI. To denote each class, the tumor volume (relative position change was not included in the class designation criteria) of both CTV and PG ROIs was taken into account, based on our previous observation that significant volumetric alterations (over 20%) are expected after week 3 [8]. Consequently, the 40 total patients were divided into two classes (in a binary fashion): Class 1 which included 19 patients (~48%) who presented a significant volume change (>20%) CTV and/or PG ROIs after the 3rd week of treatment sessions (i.e., in CBCT4) and; Class 2 which incorporated 21 patients (~52%) who displayed smaller anatomical structure variations (<20%) (Figure 1). 

### 2.3. Feature Extraction

For the CBCT scans per patient, 3DSlicer and pyradiomics were utilized to facilitate image feature calculation from the CTV and PG 3D ROIs [32,33]. As a baseline, the first CBCT feature value (week 1) was calculated (delta-radiomics), therefore providing a feature value as the percentage difference between the baseline and the latest measurement for each subsequent week (weeks 2–4). Image features are categorized as follows: (a) Shape features, referring to the position, shape and size characteristics of the ROIs; (b) First-order texture features, providing information about the spatial arrangement of intensities in the ROIs; and (c) Second-order texture features, representing the distribution of co-occurring values between neighboring pixels [33]. In total, 104 image features were calculated per ROI (i.e., CTV and PG), per week and per patient of which 12 were Shape, 17 were First-order and 75 were Second-order variables. The distribution of the Second-order features includes: (i) 14 Gray Level Dependence Matrix (GLDM) features that quantify the grey level values correlation between voxels, (ii) 24 Gray Level Co-occurrence Matrix (GLCM) features that represent the frequency that gray level value pairs with the same distance in the image appear within a ROI, (iii) 16 Gray Level Run Length Matrix (GLRLM) features that express the number of voxels in a row with the same gray level value, (iv) 16 Gray Level Size Zone Matrix (GLSZM) features that quantify the grey level zones in the ROI (with Grey level zone representing the number of voxels with the same grey value in the ROI) and (v) 5 Neighboring Gray Tone Difference Matrix (NGTDM) features that quantify the difference in the gray level value of a voxel in relation to the average gray level value in its neighbors (Table 1). A detailed description of each 104 features of the 7 feature families can be found in [33].

### 2.4. Feature Selection

**Figure 2:**
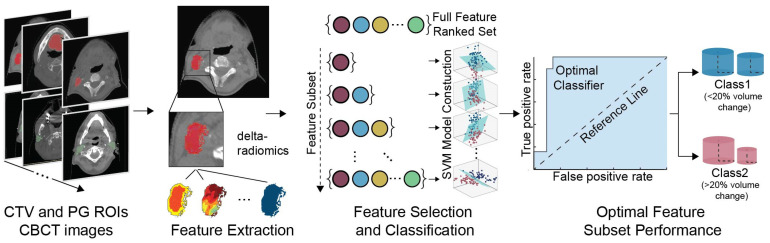
A schematic of the proposed framework workflow. CBCT images from CTV and PG ROIs are employed to calculate delta-radiomics features. The features from both ROIs are then fed into a feature selection and classification scheme to identify the feature subset with the highest discrimination power and assess overall performance.

In view of our hypothesis that image-related features could provide indications of anatomical changes at an early stage, binary classification was employed utilizing the features that corresponded to the second week of CBCT (i.e., after the first week of treatment) to discriminate between the significant (Class 1) and minor (Class 2) structural CTV and/or PG alterations. However, due to the large number of features compared to the number of samples, feature selection was employed to mitigate potential overfitting and remove redundant features. As a general methodological procedure, feature selection was applied to both CTV and PG features to isolate early anatomical information features and improve the classification performance and highlight the importance of the features selected. A schematic of the proposed framework is presented in Figure 2.

To that end, a Feature Elimination Method with Correlation Bias Reduction (RFE-CBR) method was utilized [34]. Specifically, RFE-CBR is a backward elimination feature selection approach that applies an internal linear Support Vector Machine (SVM) classifier to estimate each feature’s impact on the overall classification. As such, the internal SVM splits the classes while maintaining a maximum margin between the hyperplane and the example points [35] (more details regarding SVM can be found in the Section 2.5). RFE-CBR utilizes the normal vector to the hyperplane as a feature-ranking criterion, recursively removing minimal evaluated features from the total feature space, starting from a full set. Furthermore, RFE-CBR addresses linear dependability issues by assessing the correlation between the features, thus alleviating correlation bias in the classification results. After the end of all repetitions, an RFE-CBR ranked feature set (based on the features’ prominence) was produced by rearranging all the features in the reverse order of exclusion.

### 2.5. Classification

The subsequent classification process was performed by adopting a linear SVM classification design. SVM is a supervised-learning machine learning method to find the optimal hyperplane in an N-dimensional space [35]. In detail, SVM maps the data points (features) into multi-dimensional space taking into account the categories (classes) each data point belongs to. Then, the optimal decision boundary (hyperplane) is calculated, being the one that maximizes the distance between the two formed groups assuming linear separability. To do so, the set of weights (normal vectors) are calculated for each feature, under the premise that their linear combination predicts the class value. By maximization of the decision boundary (utilizing Lagrange multipliers) the number of nonzero weights is significantly reduced, thus resulting in a small subset of training samples that correspond to nonzero weights (support vectors) to specify the decision function. The SVM models then can be applied to new data points (examples), estimating the class they relate to by mapping them into the same space and determining the side of the hyperplane they appear. On this premise, by splitting the data into a training and a testing set, the SVM models can be built (utilizing the training set) and subsequently validated (employing the testing set). 

In the present study, feature selection and subsequent classification were employed as a Leave-One-Out Cross Validation (LOOCV) procedure to identify subject-invariant features and thus evaluate their performance towards early anatomical change discrimination. LOOCV involves allocating all-but-one instances in a training set, while the excluded data are considered a testing set. This process is repeated until all data are selected as a testing set. In the feature selection processes, RFE-CBR was applied to the training sets, thus resulting in 40 ranked feature sets. The final ranked feature sets were defined by the most frequently shared features of all folds, determined by the subsequent classification procedure. In turn, classification included the development of the SVM model based on the training and then the constructed model was evaluated in the testing set. Classification performance (i.e., the classification metrics including accuracy) was estimated as the average across all folds. As such, each SVM model would repetitively assess the classification accuracy, adding in each iteration the next RFE-CBR ranked feature to the feature subset, starting from a null set. Consequently, an optimal feature subset (the one that provided the highest accuracy) was generated. To handle the minor class imbalance, the weighted accuracy was employed as the evaluation metric, although it is presented in the following sections as accuracy to avoid readers’ confusion. Moreover, in order to assess that no overfitting or selection bias was involved in the overall classification processes, complementary 1000 classifications with random class label permutations were implemented under the same LOOCV design. Accordingly, a *p*-value was estimated as the ratio of permutations that outperformed the one obtained by the original samples to the number of total permutations [36]. All feature selection and classification algorithms were implemented using MATLAB 2020b (Mathworks Inc., Natick, MA, USA).

## 3. Results

### 3.1. Classification Performance

The highest performance achieved (in terms of prediction accuracy) was 0.90 accuracy (*p* < 0.01), 0.95 sensitivity and 0.86 specificity (Table 2) employing 19 features. Of the total of 19 features, 13 were calculated from the CTV and 6 features from the PG ROI. Interestingly, the associated machine learning model involved 5 out of 7 feature families (with the optimal subset incorporating features from all but the Shape and NGTDM families), while 3 features were common in both ROIs (i.e., Gray Level Non-Uniformity—GLDM feature family, Gray Level Non-Uniformity—GLSZM feature family, Correlation). Although the descriptions of all the estimated features used in this study are found in [33], a narrative of the selected features is presented in Table 3 and Table 4, as well as the ranking for the RFE-CBR procedure.

### 3.2. Delta-Radiomics Features

**Figure 3:**
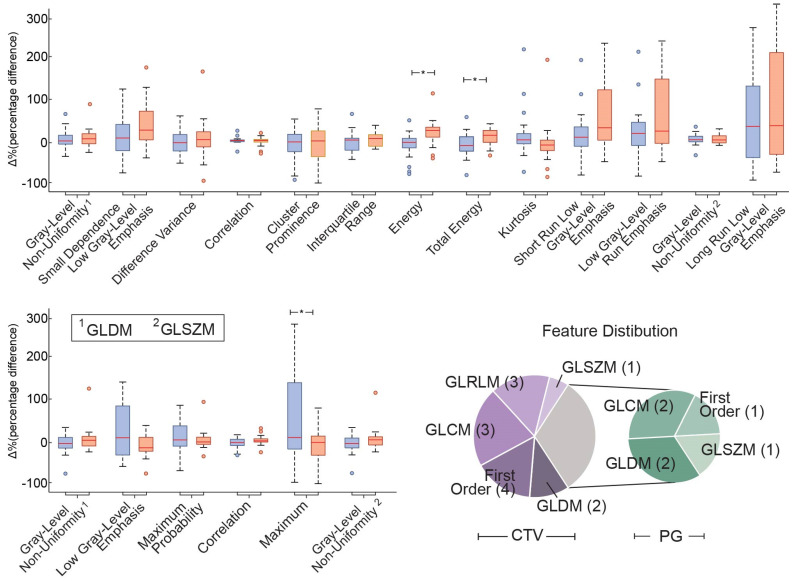
The radiomics feature incorporated in the optimal subset. The red central horizontal line in each box indicates the mean value, with the edges designating the 25th and 75th percentile, while the whiskers extend to the most extreme data points. Outliers are marked with the “o” symbol, whereas “*” indicated the significant ANOVA *p*-value < 0.05. Regarding the Gray−Level Non−Uniformity feature case, the number over the feature names indicates the different feature families with 1 being GLDM and 2 GLSZM. The pie charts represent the distribution of the PG and CTV ROIs features with respect to the feature families.

For the purpose of investigating the selected feature properties with respect to the algorithmic prediction, further analysis was conducted on the value difference of the two classes and the features’ interrelationship with the two ROIs’ volume fluctuations. As such, the analysis of the optimal delta-radiomics features revealed an increasing trend of the feature values relevant to the two classes (Figure 3). Specifically, the majority of the features (11 out of 13 CTV and 4 out of 6 PG ROI features) presented higher values in the significant alteration group (Class 2) compared to the non-significant one (Class 1). By contrast, the features “Correlation”, “Kurtosis” for CTV and “Maximum probability”, “Maximum” for the PG ROI displayed overall decrements. To that end, a one-way ANOVA between the two classes was performed on the individual features to provide indications as to the overall feature significance in the classification paradigm. However, none of the selected features presented a significant ANOVA *p*-value (<0.05), with the exception of First-order texture features “Energy” and “Total Energy” for CTV and “Maximum” for the PG ROI.

### 3.3. Model Stability Evaluation

In order to evaluate the model stability and the selected features’ robustness, additional classification was performed employing the selected CTV and PG ROIs after the completion of weeks 2, 3 and 4 of treatment (i.e., CBCTs 3, 4 and 5, respectively). In this regard, the classification accuracy, sensitivity and specificity were assessed per treatment week (Table 5). It is worth noting that although the proposed framework achieved over 0.72 accuracy regardless of the week of treatment (even after the week 3 landmark), the overall model efficiency deteriorates with respect to the early (week 1) prediction, indicating that the selected features’ values display significant variations over the course of RT treatment.

## 4. Discussion

In the current literature, adaptation in radiotherapy for head and neck cancer seems quite timely and promising, especially in the era of image-guided radiotherapy. In a review study, Avgousti et al. [37] evaluating 85 articles related to adaptive RT as an attempt to classify criteria for adaption, reported that the current thresholds which lead to replanning might be anatomical deviations > 1 cm in the external contour, average weight loss > 10%, violation in the dose coverage of the targets > 5%, and violation in the dose of the peripherals. However, beyond the expert opinion, a machine learning procedure seems more than necessary for routing clinical practice. In the current study, a machine learning framework was proposed for the early prediction of possible anatomical alterations, necessitating treatment planning adaptation. In this regard, feature selection and classification methods were applied, employing CBCT image-based radiomic features in HN cancer patients in order to predict significant volumetric changes in PG and/or CTV ROIs during RT treatment. Using appropriate features of the CBCT images acquired after the completion of the first week of RT treatment the proposed SVM model displayed high prediction accuracy of possible volumetric changes after the 3rd week of RT treatment while prominent image-related attributes that could potentially reflect RT effects regarding tumor and OARs’ characteristics were identified. 

Concerning classification performance, the optimal SVM model displayed 0.90 accuracy (0.95 sensitivity, 0.86 specificity), utilizing a small number of PG and CTV ROIs CBCT image properties. In this context, it should be noted that more recent approaches (e.g., deep learning classifiers) could theoretically achieve higher performance results. However, deep learning methods encode the information in a way that is exceedingly difficult to interpret, in the sense that the improved classification performance is achieved by providing “higher-level abstractions” of the original data [38,39]. Therefore, the features that actually provide the class discrimination tend to be rather distanced from the original features. Our objective was not only to obtain high classification accuracy but also to provide indications of the radiomic variables that can early predict ART necessity and thus, we opted for using more conventional classification methods. Furthermore, additional classification methods were applied (i.e., k-NN, gaussian SVM, LDA and Random Forest classifiers) to calculate the overall optimal performance. However, all of the aforementioned classifiers were inferior to the linear SVM (in terms of classification accuracy) and thus result comparisons were omitted. 

Moreover, the efficiency of the methodological scheme was further assessed by the low *p*-values of the permutation tests, it should be pointed out that the estimation of a global model irrespective of the RT week is exceedingly challenging. To that end, the subsequent evaluation of the selected CTV and PG ROIs features calculated after the completion of weeks 2–4 RT treatment (CBCTs 3–5), displayed significant deterioration in classification performance. On this premise, it can be inferred that the effects of radiation have a large impact on the extracted parameters. In fact, evidence suggests that HN patients that undergo RT may be at risk of experiencing oral mucositis and mucosal edema of pharyngeal and laryngeal walls due to induced glandular hyperemia, affecting gray values and thus the calculated image features [29,40,41]. It is important to note that the nature of the feature selection method considers feature importance based on the overall performance and may not directly associate with the underlying RT treatment volumetric alterations. In this regard, the optimal subset obtained might be a result of unrelated noise reduction and subsequently classification accuracy improvement [42]. Taking this into account, the high feature variability between the various subject renders a global model estimation more difficult. As such, statistical tests revealed significant differences in only a small proportion of the total feature number (3 out of 19). Furthermore, subsequent analysis was performed on the associations between the CTV and PG tumor volumes and the variables incorporated in the optimal feature subset (via Pearson correlation) revealed that the selected features demonstrate linear dependence with the CTV/PG volumes only to a small degree (testing the null hypothesis of no relationship between the observed phenomena (*p*-value smaller than the significance level of 0.05).

From this standpoint, the absence of a clear overall trend, suggests that the tumor(s) and PGs respond to the RT treatment in a non-systematic patient-specific manner. Therefore, since the input of the adopted feature selection procedure originated only from features after the completion of week 1 (CBCT 2), the decrements in the classification accuracy could be explained as a result of therapy-related feature value modifications. In this regard, different input (e.g., for week 2) might result in a different subset elucidating additional image-related characteristics that could achieve better differentiation properties in a later RT stage. However, the scope of this study was to provide a framework for early prediction, while in parallel illustrating the specific image attributes that correspond to RT-related volumetric changes. Nevertheless, the fact that the selected features (estimated in the various weeks) display high classification performance (Table 5) even after the 3rd-week landmark, denotes their prominence as efficient indicators of RT-related anatomical alterations.

Regarding the image-based delta-radiomic features incorporated in the optimal subset, only a small fraction of the overall 208 (104 for each ROI) was selected as being the most discriminative, reducing the complexity of the generated model and implying that no overfitting occurred during the feature selection and classification processes. As such, most of them (13 out of 19) originate from the CTV ROI with the PG ROI features representing the minority group (6 out of 19). This could be attributed to the fact that the tumor ROI is inside the high dose region during RT cycles, leading to texture variations for the majority of cases while PGs are more protected in contrast to tumor ROI. In this context, it could be conjectured that the employment of only the CTV ROI features might lead to similar classification performance without the need for a 2-ROI intricate design of the machine learning framework. However, this might result in substantial information loss in relation to the tumor coverage that accounts for OARs’ radiation overdose [4]. On the other hand, in the event of applying feature selection on CTV and PG features separately (with the purpose of disentangling the two ROIs) and then combining the ensuing subsets, would not account for potential bias due to the high correlation of the corresponding groups, leading to the development of unreliable classification models [43].

In addition, the individual features integrated into the optimal subset belong to 5 (out of 7) feature families with only shape and NGTDM being excluded. Although this does not denote that usable information cannot be extracted from the latter categories, it implies decreased differential characteristics compared to the other feature families that could result from tumor shape irregularities, high variance and reduced neighboring gray level difference [44]. Nonetheless, the GLCM feature family includes the “Difference Variance”, “Maximum Probability”, “Correlation” (selected in both CTV and PG ROIs) and “Cluster Prominence” features, suggesting distinct aspects of spatial gray-level variation within local neighbors on a pixel basis. On this premise, the GLCM “Cluster Prominence” feature could support early discrimination as it provides an intensity variability measure that can detect small intensity differences between image voxels [45]. Furthermore, “Difference Variance” has been consistently reported to be utilized as a measure of the heterogeneity in an ROI [46]. Consequently, its discriminative ability is in accordance with previous studies in which “Difference Variance” was found to be prognostic for local tumor control in HN cancer patients treated with chemo-radiotherapy [47]. In the same manner, “Correlation” indicates a local gray-level dependency on the texture ROI. It has been reported to be able to differentiate between benign and malignant solitary pulmonary nodules in lung cancer [48], while a previous study (regarding the PG) reported that the correlation value appeared to be decreased for an irradiated gland compared to a normal one [30]. Additional GLCM characteristics have also been reported in other types of cancer. For example, “Maximum Probability” has been considered an effective noninvasive predictive biomarker for a pathological response from chemoradiation before surgery for Non-Small Cell Lung Cancer patients [49]. Nonetheless, there is scarce evidence for a reliable interpretation of the GLCM implications in the context of HN early anatomical alterations, necessitating further future research [50]. The “Grey Level Non-Uniformity” feature was also designated as important from both CTV and PG ROIs in two feature families (i.e., GLDM and GLSZM). Interestingly, in both cases, the mean (and median) value after week 1 of the significant alterations (class 2) is higher than the non-significant (class 1). Although no statistical difference was found, this fact corroborates with other studies showing that smaller “Grey Level Non-Uniformity” values display higher uniformity and therefore suggesting that “Grey Level Non-Uniformity” represents the response to cancer treatment as regards to radiotherapy [51]. As a result, other research works have proposed the employment of CT-image “Grey Level Non-Uniformity” as a predictive marker for adaptive radiotherapy and for local failure (disease persistence or reappearance) in HN cancer patients [28,52]. In a similar fashion, “Kurtosis” measures the peakedness of the distribution values and the complexity of the organizational structure and tumor heterogeneity. Congruent with recent studies, our results demonstrated higher “Kurtosis” in the non-significant patient group as increased values are correlated with more homogenous ROIs, which tend to have a better response to radiotherapy [23,53]. 

While the proposed framework in the current study displays high performance, the overall reliability of the selected features should be considered with caution. The key concern is that the extracted values of features might be influenced by the data acquisition and handling [54]. This is also illustrated in previous works that study the radiomics’ potential in order to enhance clinical decision-making, suggesting that the multiple steps related to image acquisition, pre- and post-processing and feature extraction can affect the radiomics computed values and thus the results’ reproducibility [55]. In this regard, we opted to avoid utilizing absolute feature values, thus obtaining the different patient image properties by comparing the delta-radiomics percentage alterations of the baseline CBCT image (CBCT 1) to the following week’s images, using the same acquisition parameters. As such, the acquired CBCT images during the course of RT treatment had the same field of view, kVp, slice thickness, etc., as the baseline.

## 5. Conclusions

In this study, an early predictive machine learning model was implemented for the identification planning adaptation requirement due to possible anatomical changes for HN cancer patients treated with RT. The proposed framework was successful in obtaining high classification performance from the 1st week of treatment. Moreover, it was able to designate important CBCT image-based features, providing insights into the radiomic characteristics related to significant tumor-volume deviations and thus paving the way for a more rigorous, fast and cost-effective adaptive RT planning.

## Figures and Tables

**Table 1:** Delta Radiomics Feature Families.

Feature Family	Number of Features	Description
Shape	12	Descriptors of the two/three-dimensional size and shape of the ROI. Gray level intensity distribution in the ROI does not affect these feature values.
First Order	17	Describes the distribution of grey values within the image region.
GLDM	14	Quantifies gray level dependencies in an image. A gray level dependency is the number of connected voxels within a specific distance that are dependent on the center voxel.
GLCM	24	Represents the frequency that gray level value pairs with the same distance in the image appear within an ROI.
GLRLM	16	Quantifies gray level runs. Run is the length in the number of pixels, of consecutive pixels that have the same gray value.
GLSZM	16	Quantifies gray level zones in an image. A gray level zone is the number of connected voxels that share the same gray level value.
NGTDM	5	Quantifies the difference between a voxel’s gray value and the average gray value of its neighbor voxels within a specific distance.

**Table 2:** Classification Performance Results.

Accuracy	Sensitivity	Specificity	F1-Score	Area Under the Curve
0.90 **	0.95	0.86	0.90	0.91

Note: Asterisks (**) marks the permutation significance testing (1000 permutations). ** *p* < 0.01.

**Table 3:** The CTV ROI Features Incorporated in the Optimal Classification Model.

Feature	Feature Family	Equation	Ranking	Definition
Gray Level Non Uniformity	GLDM	$GLN=\frac{\sum_{i=1}^{N_{g}}{\left(\;\sum_{j=1}^{N_{d}}P\left(i,j\right)\right)}^{2}}{N_{z}}$	(15)	Quantifies the gray level intensity values similarity in the image. A lower GLN value implies a greater similarity in intensity values
Small Dependence Low Gray Level Emphasis	GLDM	$SDLGLE=\frac{\sum_{i=1}^{N_{g}}\;\sum_{j=1}^{N_{d}}\frac{P\left(i,j\right)}{i^{2}j^{2}}}{N_{z}}$		Lower gray-level values imply a joint distribution of small dependence.
Difference Variance	GLCM	$DV=\overset{N_{g}-1}{\underset{k=0}{\sum }}(k-DA^{2})p_{x-y}\left(k\right)$	(8)	Measures the heterogeneity. Higher weights on differing intensity level pairs deviate more from the mean
Correlation	GLCM	$Cor=\frac{\sum_{i=1}^{N_{g}}\sum_{j=1}^{N_{g}}p\left(i,j\right)ij-\mu_{x}\mu_{y}}{\sigma_{x}\left(i\right)\sigma_{y}\left(j\right)}$	(4)	Quantifies the linear dependence of gray level values to their respective voxels in the GLCM
Cluster Prominence	GLCM	$cl\;pr=\overset{N_{g}}{\underset{i=1}{\sum }}\;\overset{N_{g}}{\underset{j=1}{\sum }}{\left(i+j-\mu_{x}-\mu_{y}\right)}^{4}p\left(i,j\right)$	(16)	Quantifies the skewness and asymmetry of the GLCM. Lower values imply lower asymmetry about the mean.
Interquartile Range	FIRST ORDER	$int\;ran=P_{75}-P_{25}\;\;$	(19)	Difference between percentiles of the image array
Energy	FIRST ORDER	$en=\overset{N_{p}}{\underset{i=1}{\sum }}{\left(X\left(i\right)+c\right)}^{2}$	(3)	Measures the magnitude of voxel values in an image. Larger values show a greater sum of the squares of these values
Total Energy	FIRST ORDER	$t\;en=V_{voxel}\;\overset{N_{p}}{\underset{i=1}{\sum }}{\left(X\left(i\right)+c\right)}^{2}$	(7)	Is the value of energy feature scales by the volume of the voxel in cubic mm
Kurtosis	FIRST ORDER	$kurt=\frac{\frac{1}{N_{p}}\sum_{i=1}^{N_{p}}{\left(X\left(i\right)-\bar{X}\right)}^{4}}{{\left(\frac{1}{N_{p}}\sum_{i=1}^{N_{p}}{\left(X\left(i\right)-\bar{X}\right)}^{2}\right)}^{2}}$	(18)	Measures the ROI’s distributions of values peakedness. The mass of the distribution is concentrated towards the tail(s) for higher kurtosis values.
Short Run Low Gray Level Emphasis	GLRLM	$SRLGLE=\frac{\sum_{i=1}^{N_{g}}\;\sum_{j=1}^{N_{r}}\frac{P(i,j|\theta )}{i^{2}j^{2}}}{N_{r}\left(\theta \right)}\;$	(17)	Measures the joint distribution for shorter run lengths with smaller gray level values
Low Gray Level Run Emphasis	GLRLM	$LGLRE=\frac{\sum_{i=1}^{N_{g}}\;\sum_{j=1}^{N_{r}}\frac{P(i,j|\theta )}{i^{2}}}{N_{r}\left(\theta \right)}$	(11)	Measures the distribution of low gray level values. Higher values indicate greater concentration of low gray level values in the image
Gray Level Non Uniformity	GLSZM	$GLN=\frac{\sum_{i=1}^{N_{g}}{\left(\;\sum_{j=1}^{N_{s}}P\left(i,j\right)\right)}^{2}}{N_{z}}$	(2)	Measures the distribution of large area size zones. Greater value indicative larger size zones and more coarse textures
Long Run Low Gray Level Emphasis	GLRLM	$SRHGLE=\frac{\sum_{i=1}^{N_{g}}\;\sum_{j=1}^{N_{r}}\frac{P(i,j|\theta )}{i^{2}}j^{2}}{N_{r}\left(\theta \right)}$	(14)	Quantifies the joint distribution of shorter run lengths with higher gray level values

**Table 4:** The PG ROI Features Incorporated in the Optimal Classification Model.

Feature	Feature Family	Equation	Ranking	Definition
Gray Level Non Uniformity	GLDM		(10)	Same as (Gray Level Non Uniformity)
Low Gray Level Emphasis	GLDM	$LGLE=\frac{\sum_{i=1}^{N_{g}}\;\sum_{j=1}^{N_{d}}\frac{P\left(i,j\right)}{i^{2}}}{N_{z}}$	(12)	Measures the distribution of low gray level values. Higher values indicate greater concentration of low gray level values in the image
Maximum Probability	GLCM	maxprob=max(p(i,j))	(6)	Measures the occurrences of the most predominant pair of neighboring intensity values
Correlation	GLCM		(1)	Same as (Correlation)
Maximum	FIRST ORDER	max=max(X)	(9)	The maximum gray level intensity within the ROI
Gray Level Non Uniformity	GLSZM		(5)	Same as (Gray Level Non Uniformity)

**Table 5:** Model Performance Per Week of Treatment.

Week/CBCT	Accuracy	Sensitivity	Specificity
1/2	0.90 **	0.95	0.86
2/3	0.85 **	0.86	0.84
3/4	0.75 *	0.76	0.74
4/5	0.72 *	0.73	0.72

Note: Asterisk (*) marks the permutation significance testing (1000 permutations). * *p* < 0.05; ** *p* < 0.01.

## Data Availability

The data presented in this study are available on request from the corresponding author. The data are not publicly available due to patient confidentiality restrictions.
